# Structural basis for 5'-ETS recognition by Utp4 at the early stages of ribosome biogenesis

**DOI:** 10.1371/journal.pone.0178752

**Published:** 2017-06-02

**Authors:** Fabiola R. Calviño, Markus Kornprobst, Géza Schermann, Fabienne Birkle, Klemens Wild, Tamas Fischer, Ed Hurt, Yasar Luqman Ahmed, Irmgard Sinning

**Affiliations:** Heidelberg University Biochemistry Center (BZH), Im Neuenheimer Feld 328, Heidelberg, Germany; University of Edinburgh, UNITED KINGDOM

## Abstract

Eukaryotic ribosome biogenesis begins with the co-transcriptional assembly of the 90S pre-ribosome. The ‘U three protein’ (UTP) complexes and snoRNP particles arrange around the nascent pre-ribosomal RNA chaperoning its folding and further maturation. The earliest event in this hierarchical process is the binding of the UTP-A complex to the 5'-end of the pre-ribosomal RNA (5'-ETS). This oligomeric complex predominantly consists of β-propeller and α-solenoidal proteins. Here we present the structure of the Utp4 subunit from the thermophilic fungus *Chaetomium thermophilum* at 2.15 Å resolution and analyze its function by UV RNA-crosslinking (CRAC) and in context of a recent cryo-EM structure of the 90S pre-ribosome. Utp4 consists of two orthogonal and highly basic β-propellers that perfectly fit the EM-data. The Utp4 structure highlights an unusual Velcro-closure of its C-terminal β-propeller as relevant for protein integrity and potentially Utp8 recognition in the context of the pre-ribosome. We provide a first model of the 5'-ETS RNA from the internally hidden 5'-end up to the region that hybridizes to the 3'-hinge sequence of U3 snoRNA and validate a specific Utp4/5'-ETS interaction by CRAC analysis.

## Introduction

**Fig 1:**
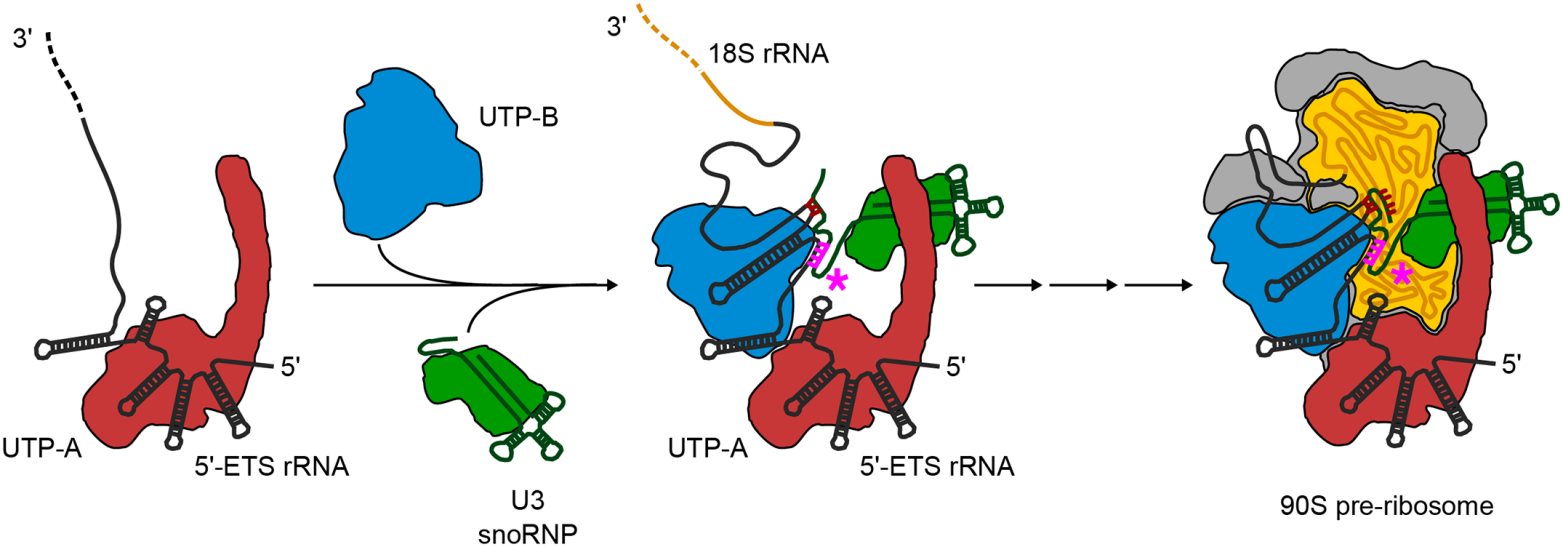
Model of co-transcriptional assembly of the 90S pre-ribosome. The nascent 5'-ETS (black line) recruits the early 90S modules (UTP-A, UTP-B, and U3 snoRNP) in a hierarchical fashion, with the UTP-A complex being the first one that binds to the extreme 5'-end of the pre-rRNA. This early assembly intermediate, together with the subsequently transcribed pre-18S rRNA (yellow line) and additional factors, forms the 90S pre-ribosome. Complexes are labeled accordingly. The 3'-hinge region is highlighted in pink. Figure is adapted from [9].

The ribosome is the cellular machinery responsible protein synthesis and therefore is vital to cellular function. Assembly in prokaryotes is less complex and to a certain degree spontaneous, while ribosome biogenesis in eukaryotes is a hierarchical and elaborate process. It involves more than 200 non-ribosomal protein factors, which act at different steps of maturation [1, 2]. Several pre-ribosomal intermediates of the small (pre-40S) and large subunit (pre-60S) have been identified, which are eventually exported to the nucleus and further on to the cytoplasm for final processing and assembly of the mature 80S ribosome. The earliest intermediate is the huge 90S pre-ribosome or small-subunit processome (SSU) [3, 4] that is co-transcriptionally assembled in the nucleolus by incorporation of several modular subcomplexes onto the nascent pre-rRNA (Fig 1). This primary transcript, called 35S rRNA in yeast, carries the mature 5.8S, 18S and 25S rRNA sequences [4], which are separated by internal (ITS) and external transcribed spacer (ETS) RNA elements that are removed by multiple rRNA processing steps. The early rRNA cleavages occurring during 90S particle biogenesis lead to removal of the 5' terminal ETS rRNA (5'-ETS) and also separate the biogenesis pathways of pre-40S and pre-60S particles. The approximately 70 assembly factors present in the 90S pre-ribosome have been assigned to several complexes involved from the very beginning of 90S particle assembly [3]. Among these are the two large ‘U three protein’ UTP-A and UTP-B complexes as well as the Mpp10-Imp3-Imp4 complex [5] and the U3 snoRNP [3, 4]. The UTP-A complex is presumably the earliest complex that associates with the nascent 35S rRNA [6] and binds to the 5'-end of the 5'-ETS up to the binding site for the U3 3'-hinge (Fig 1). Although the composition of UTP-A varies between different organisms, it contains at least the six conserved subunits Utp4, 5, 8, 10, 15, and 17 [7]. The UTP-A subunits form an interconnected protein-protein network and contain several β-propeller and α-helical domains. The UTP-A subunits have been shown to be essential for biogenesis of both the 40S and 60S subunit and have been linked to pre-rRNA transcription and are hence also termed t-UTP proteins [8]. A recent cryo-EM study of the entire 90S particle isolated from the thermophilic fungus *Chaetomium thermophilum* has revealed the overall architecture of the 90S pre-ribosome [9]. Within this cryo-EM structure, UTP-A was identified at the base of the 90S particle bound to the first helices of the 5'-ETS, which was however not modeled.

A number of diseases resulting from defective ribosome biogenesis have been described in the literature [10, 11] with some of them being related to the earliest assembly steps. Thus, a single point mutation (R565W) in the human Utp4 homolog is known to cause Native American Indian Childhood cirrhosis (NAIC) [10]. In yeast, no phenotype could be observed upon introduction of the corresponding mutation into Utp4, but deletion of C-terminal residues abolished the binding to Utp8 in a yeast-2-hybrid assay. These truncations also exhibited reduced levels of 18S and 25S rRNA, indicative of defects in ribosome biogenesis.

In order to shed light on the structure-function relationship of Utp4 in early ribosome biogenesis, we solved the crystal structure of Utp4 from the thermophilic fungus *Chaetomium thermophilum* (*ct*) at a resolution of 2.15 Å and analyzed it in the context of a previously published 90S pre-ribosome cryo-EM structure [9]. Combining structural data with UV-RNA crosslinking and including previous biochemical data we generate a model of the 5'-ETS that provides insights into the molecular interactions of Utp4 with RNA and Utp8, with implications for the Utp4 structure for the entire fold of the UTP-A complex and for the assembly of the 90S pre-ribosome.

## Material and methods

### Expression and purification of Utp4

Cloning of Utp4 from *Chaetomium thermophilum* was described previously [12]. Expression of native Utp4 from *Chaetomium thermophilum* (Uniprot CTHT_0058380, residues 1–848) was carried out in *E*. *coli* Rosetta 2 (DE3) cells in ZYM5052 medium, grown at 37°C to an OD_600_ of 1.0. Then, the temperature was dropped to 23°C and the cells were grown further overnight. After harvesting, the cell pellet was mixed with lysis buffer (20 mM HEPES pH 8.0, 250 mM NaCl, 20 mM MgCl_2_, 20 mM KCl, 40 mM imidazole) and lysed in a M-110L Microfluidizer (Microfluidics). Lysate was clarified by centrifugation (20000 ×g, 20 min, 277 K) and the filtered supernatant was loaded onto a 5 ml HisTrap FF column (GE Healthcare) previously equilibrated with 10 column volumes (CV) of lysis buffer. The column was further washed with 10 CV of lysis buffer and the protein was eluted using 5 CV of wash buffer containing 500 mM imidazole. Elution fractions containing Utp4 were concentrated and further purified by size exclusion chromatography (SEC). The sample was loaded onto a Superdex 200 26/60 prep grade column (GE Healthcare) equilibrated with SEC buffer (20 mM HEPES pH 7.5, 200 mM NaCl, 20 mM MgCl_2_, 20 mM KCl, 1 mM DTT). Se-Met labelled Utp4 was expressed as the native protein using a modified protocol for the inhibition of methionine-biosynthesis with 0.5 mM IPTG induction [13].

### Co-expression of the UTP-A complex and transcription of the 5'-ETS

Heterologous co-expression of the *Chaetomium thermophilum* UTP-A complex in yeast strain W303 (*Matα*, *ade2-1*, *his3-11*, *15*, *leu2-3*,*112*, *trp1-1*, *ura3-1*, *can1-100*) was carried out as previously described using a high copy vector system (2μ, *TRP1*, *LEU2*, and *URA3* marker, respectively) that allows *GAL1-10* promoter-driven expression of up to six different proteins [12]. In addition to the co-expressed six UTP-A factors (Utp4/His_6_-Utp4, Utp5, Utp8, proteinA-TEV-Utp10, Flag-Utp15, and Utp17), the 5'-ETS (1-587 nt) was transcribed in this yeast strain from a fourth vector (*HIS3* marker) using a *GAL1* promoter, in which bases 345–472 of the promoter sequence (5'-UTR of the transcribed RNA) have been deleted. All vector plasmids are listed in S1 Table. Yeast cells were grown in raffinose (SRC-Trp-Leu-Ura-His)-containing medium to an OD_600nm_ of ~2.0, before GAL promoter was induced by addition of 2-fold concentrated galactose-containing medium (2x YPG) at a ratio of 1:1. Cells were harvested after induction for 5 hours by centrifugation at 4000 ×g, washed with dH_2_O, frozen in liquid nitrogen, and stored at −20°C until use.

Affinity purification of proteinA-TEV-tagged UTP-A factor Utp10 to isolate the UTP A/5’-ETS complex (co-expressed in yeast) was performed in a TAP buffer containing 50 mM Tris-HCl (pH 7.5), 150 mM NaCl, 1.5 mM MgCl_2_, 5% (v/v) glycerol, 0.1% (v/v) NP-40, and 1 mM DTT as previously described [14, 15].

### *In vitro* UV-induced CRAC analysis

UV-mediated protein-RNA crosslinking and cDNA deep sequencing analysis (CRAC) of His_6_-Utp4 was performed using the reconstituted and affinity-purified UTP-A/5'-ETS. This RNP carrying untagged Utp4 was used as negative control (‘no His_6_ tag’). After IgG affinity-purification of the UTP-A complex via proteinA-TEV-Utp10, elution was carried out by TEV cleavage using GST-TEV for 2 hours at 16°C. 500 μL of the corresponding TEV eluates were transferred to 6-well culture plates placed on ice and UV irradiated (254 nm) in the Stratalinker^®^ Crosslinker at 400 mJ/cm^2^. Cross-linked RNA was digested with 0.4 units of RNace-IT ribonuclease cocktail (Thermo Fisher Scientific). Subsequent Nickel-purification under denaturing conditions and all further steps were carried out as previously described [16]. The cDNA library generated by SuperScript^®^ III reverse transcription (Thermo Fisher Scientific) was deep sequenced at the DKFZ (Heidelberg) using the Illumina MiSeq system (single-end 50bp). After filtering duplicate reads using the script included in the pyCRAC program [17], reads were mapped to the 5'-ETS sequence (nts 1–587) of *Chaetomium thermophilum* using the Novoalign software. Coverage and mutation hotspots were calculated with pyCRAC, plots were generated with R scripts from the pileup files (https://www.r-project.org/).

### Crystallization, structure determination, and RNA modelling

Purified Utp4 was concentrated to 10–20 mg/mL and crystallized in an automated crystallization platform using the sitting drop vapour diffusion method. For structure determination by single anomalous dispersion (SAD) we prepared seleno-methionine labelled protein, which crystallized in a condition containing 5% (w/v) 2-methyl-1,5-pentane-diol (MPD) and 100 mM Tris-HCl pH 8.5). Prior to data collection, crystals were cryo-protected by soaking in mother liquor containing 20% (v/v) ethylene glycol and directly flash-cooled in liquid nitrogen. X-ray diffraction data were collected at the European Synchrotron Radiation Facility (ESRF) beamlines ID14-4 and ID23-2. Data was integrated with XDS [18] and scaled and merged with AIMLESS [19] from the CCP4-package [20]. An initial model of Utp4 was obtained from SAD data using the PHENIX package [21]. Iterative model building, refinement, and validation were performed with COOT [22] and PHENIX. All structural figures were prepared using PyMOL (Molecular Graphics System, Version 1.5.0.4 Schrödinger, LLC; http://www.pymol.org). 5’-ETS modelling was based on previous biochemical restraints [23], secondary structure prediction and a cryo-EM study at 7.3 Å resolution [9], and was manually done in COOT with local geometry and real space adjustments.

## Results

### Crystal structure of Utp4

**Fig 2:**
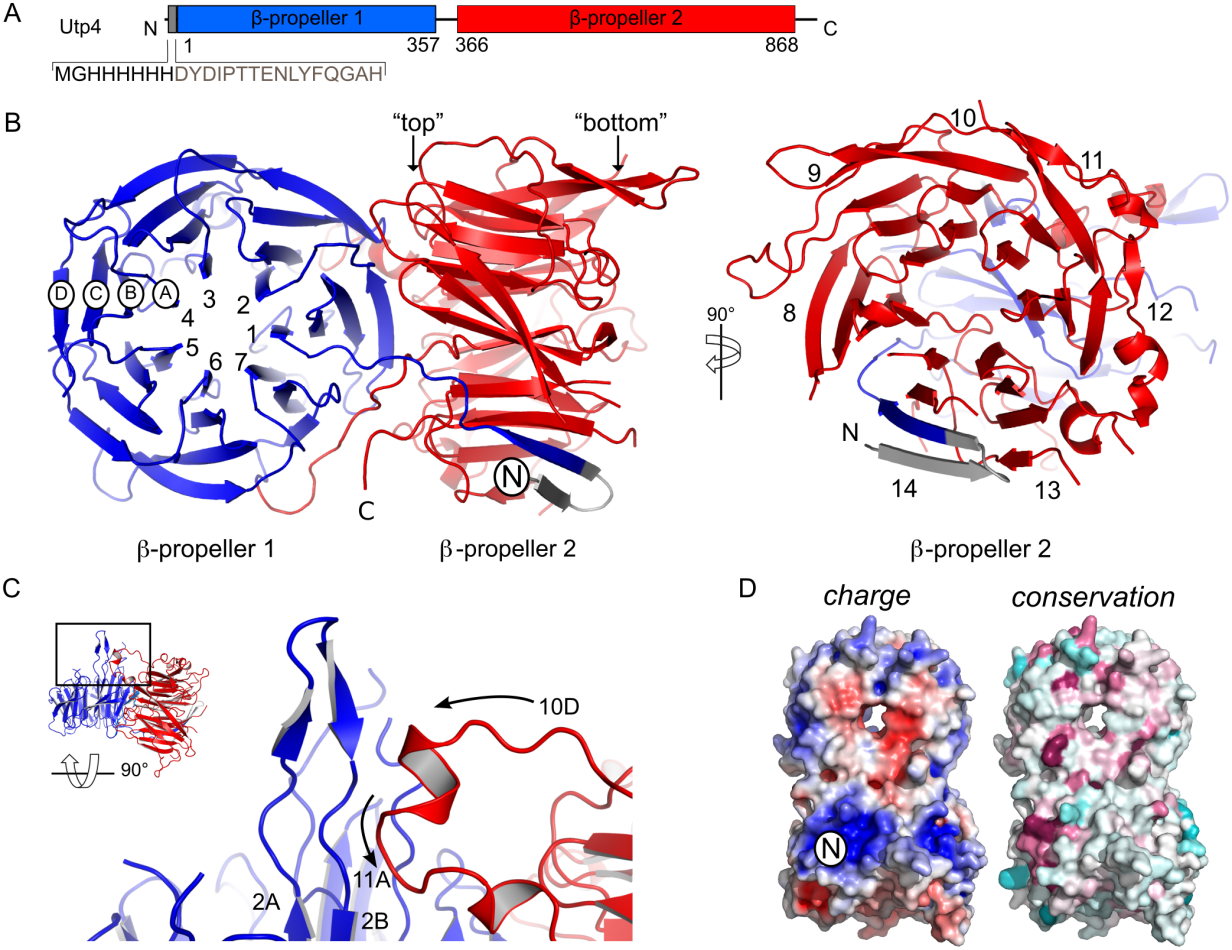
Structure of Utp4 from *Chaetomium thermophilum*. **(A)** Domain architecture of Utp4. Domains present in the crystal structure are given by residue numbers and are highlighted in colour. The N-terminal β-propeller 1 covers residues from 38 to 381 and is shown in blue and the C-terminal β-propeller 2 (residues 393 to 890) in red. (His)_6_-tag and TEV-site are represented in grey. **(B)** The overall structure of Utp4 presents two 7-bladed β-propellers in tandem. N- and C-termini are indicated and blades are numbered. Each β-blade consists of four β-strands (ABCD). **(C)** Tertiary interaction of β-propellers. A hairpin between β-strands 2A and 2B of β-propeller 1 packs against an α-helix between β-strands 10D and 11A of β-propeller 2 (view rotated by 90° in respect to **A**). **(D)** Surface charge (left panel) and conservation (right panel) of Utp4. The electrostatic surface (red: negative, blue: positive, contoured at ±5 *k*_*B*_T/e) indicates extended positively charged patches in both β-propellers. Sequence conservation mapped on the molecular surface (magenta: conserved, cyan: variable) is most pronounced around a highly positive charged patch at the N-terminus (indicated with ‘N’).

Following up on our reconstitution of entire *ct*UTP-A and *ct*UTP-B complexes from heterologously expressed components in yeast [12] we set out to determine the structure of Utp4 and to derive the molecular basis of its interactions within the 90S pre-ribosome (*ct* is always omitted in the following for clarity). UTP-A was also part of our systematic analysis of ribosome biogenesis factors from *Chaetomium thermophilum* [12]. The crystal structure of Utp4 was solved at a resolution of 2.15 Å by the single wavelength anomalous dispersion (SAD) method using seleno-methionine labelled protein (Table 1). Utp4 comprises two 7-bladed β-propellers (numbered 1 to 14) with four strands (A, B, C and D) per blade (Fig 2A and 2B). The overall architecture reveals the two propellers to be arranged in an orthogonal manner with the side of the N-terminal β-propeller 1 packing with β-strand 1D against the top of the C-terminal β-propeller 2 (S1 Fig). The arrangement is further stabilized by a lateral interaction of two protruding loops from the opposing propellers (Fig 2C). The first loop between β-strands 2A and 2B of β-propeller 1 forms an extended β-hairpin that packs against an α-helix presented by the opposing loop that emanates from β-strands 10D and 11A of β-propeller 2 (Fig 2C). Overall about 17% of the accessible surface area of the N- and C-terminal β-propellers are involved in the propeller-interaction. A long *Chaetomium thermophilum* specific C-terminal insertion between β-strands 13C and 13D (residues 717–825) is not resolved in the crystal structure (S1B Fig). The surface charge and conservation analyses of Utp4 revealed several positively charged patches that could be involved in interactions with RNA (Fig 2D). Most prominent, residues at the very N-terminus constituting the first β-strand form a basic surface patch, which is also conserved. This first β-strand pairs with the very C-terminus of β-propeller 2 by β-augmentation.

**Table 1:** Data collection and refinement statistics.

	*Native*	*Se-SAD*
**Data collection**		
Resolution range (Å)	47.53–2.15 (2.22–2.15)	47.59–2.80 (2.95–2.80)
Space group	*C*2	*C*2
Unit cell		
a, b, c (Å)	203.0 81.6 112.3	203.0 81.6 112.3
α,β,γ (°)	90 110.6 90	90 110.6 90
Unique reflections	93437 (9228)	43202 (6234)
Multiplicity	5.8 (5.8)	11.7 (11.9)
Completeness (%)	99.9 (99.7)	100 (100)
Mean I/ σ(I)	13.7 (2.3)	18.8 (6.1)
Wilson B-factor (Å^2^)	34.7	31.0
R_pim_ (%)	5.7 (52.0)	3.9 (13.7)
CC*	0.999 (0.945)	0.999 (0.957)
Anomalous completeness (%)		100 (99.5)
RCR_anomalous		1.31
FOM before DM^+^		0.353
**Refinement**		
R-work	0.1768 (0.2352)	
R-free	0.2331 (0.3083)	
Number of non-hydrogen atoms	11683	
macromolecules	11031	
water	652	
RMSD bonds, (Å)	0.008	
RMSD angles (°)	1.13	
Ramachandran plot (%)		
favored	97	
outliers	0.15	
Average B-factor (Å^2^)	39.0	
macromolecules	39.0	
solvent	38.7	

Statistics for the highest-resolution shell are shown in parentheses. R_pim_: precision-weighted merging R-factor. CC* is an estimate of the ‘true’ CC1/2 of the data under examination to the unknown true intensities. RCR_anomalous: RMS correlation ratio for anomalous data. FOM: figure of merit. DM: density modification.

^+^Values at 4.0 Å resolution cut-off.

### Velcro-closure of the C-terminal β-propeller

**Fig 3:**
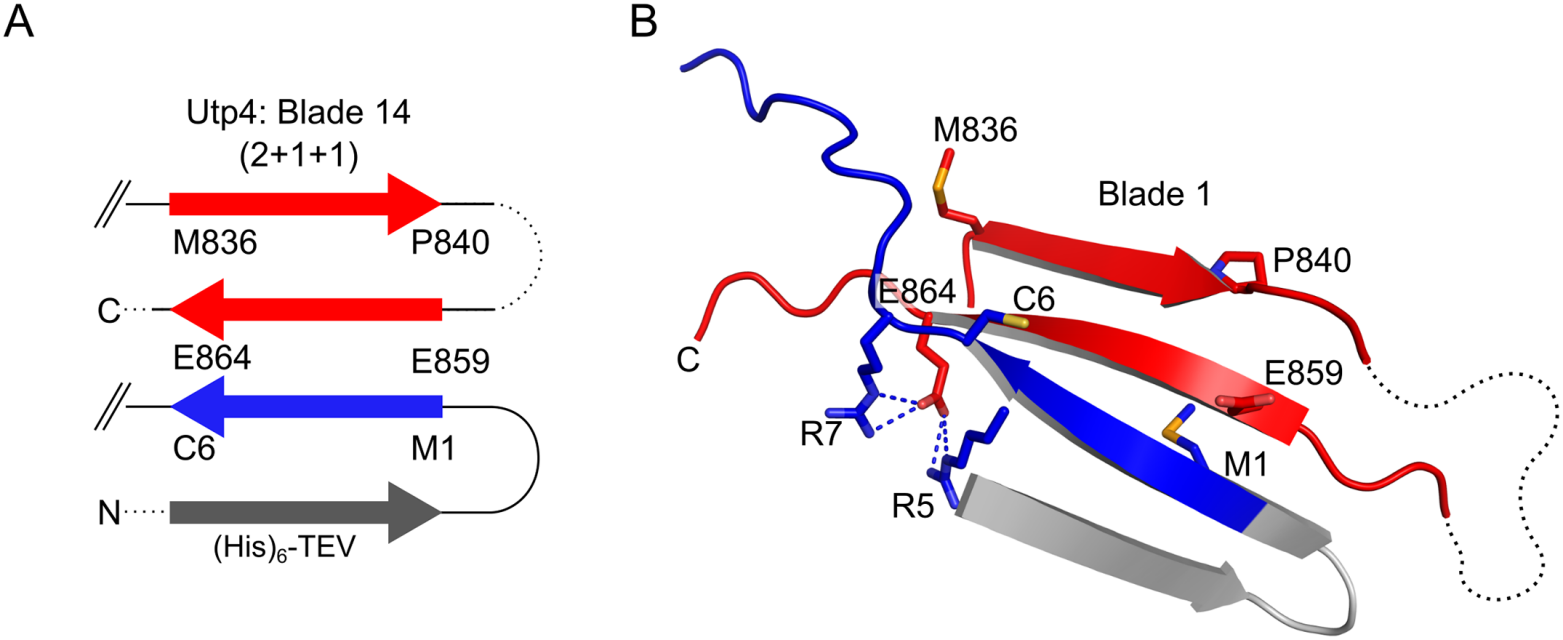
Uncommon Velcro-closure of the C-terminal β-propeller 2. **(A)** Schematic representation of the last blade 14 of Utp4. The four β-strands of the blade are represented as arrows in different colours: 14A and B (C-terminus of β-propeller 2, red), 14C (N-terminus of β-propeller 1, blue), and 14D ((His)_6_-TEV-tag, grey). **(B)** Close-up of blade 14 complemented by the very N-terminus of the polypeptide chain, forming an uncommon parallel β-strand 14C (blue) and the artificial TEV site (grey) forming an antiparallel β-strand 14D. The highly conserved residues and their hydrogen-bonding network stabilizing the blade and therefore the Velcro-closure of β-propeller 2 are represented in sticks. Salt-bridges are indicated by dashed lines.

In general, the individual blades of a β-propeller are rather labile arrangements and β-propeller proteins have evolved different ways to keep the blades within a closed ring. Most of the β-propellers circularly permute up to three β-strands of the last blade to the N-terminus of the protein in order to close the ring. This mechanism has been defined as ‘Velcro-closure’ and different combinations (3+1, 2+2, 1+3) have been described depending on the number of strands provided by each terminus of the β-propeller domain to build up the last blade [24]. For Utp4 we observe an uncommon Velcro-closure for the most C-terminal blade 14 of β-propeller 2 (Fig 3). While the N-terminal β-propeller 1 forms a continuous domain (residues 38–381), blade 14 is completed by parallel β-augmentation of the very N-terminus of Utp4 (β-strand 14C) and the N-terminal TEV cleavage site (anti-parallel β-strand 14D) introduced during cloning. The closure between the native N- and C-termini (S2 Fig) is not only stabilized by unspecific main chain interactions between the parallel β-strands 14B and 14C, but also by various side chain interactions (Fig 3B). In particular, a network of salt-bridges between conserved residues (R5-E864, R7-E864) help in stabilising the Velcro-closure suggesting a highly specific arrangement. As β-strand 14C constitutes the native N-terminus of Utp4, stable Velcro-closure *in vivo* calls for a ‘2+1+1’ blade completion *in trans* (14D as last ‘+1’ number) by another protein (see below).

### Utp4 UV-crosslinking with the 5'-ETS

**Fig 4:**
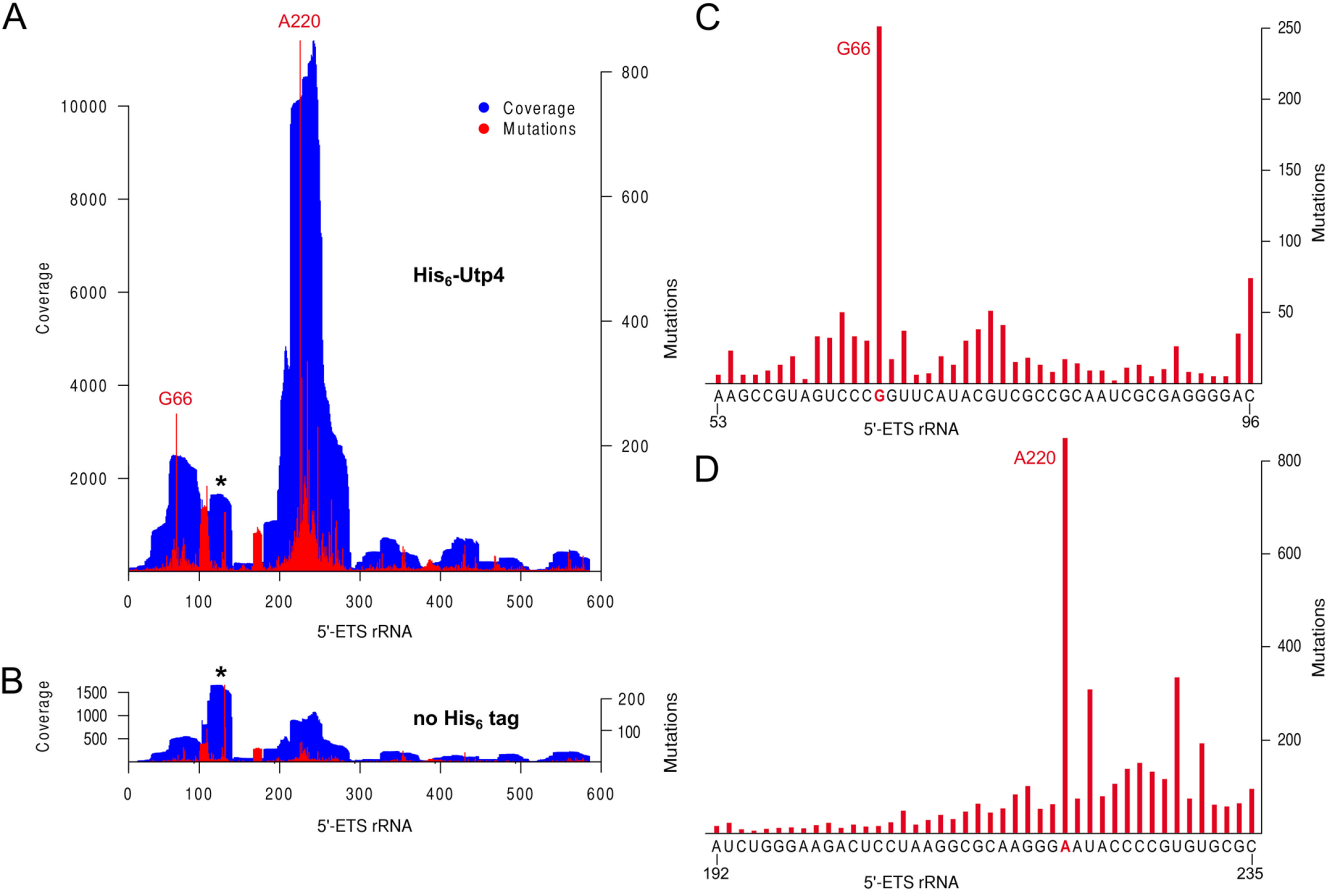
*In vitro* protein-RNA UV crosslinking analysis of *ct*Utp4. **(A)** Hits obtained from deep sequencing analysis of *Chaetomium thermophilum* His_6_-Utp4 (coverage, blue) mapped within the 5'-ETS (nucleotides 1 to 587) after UTP-A/5'-ETS RNP assembly by co-expression in yeast. **(B)** Mutations (deletions and substitutions) identified after cDNA library synthesis are indicated by red bars. Mutational hot spots observed in the two crosslinked regions are labeled accordingly (G66 and A220). As background control, the UTP-A/5'-ETS complex carrying untagged Utp4 (“no His_6_ tag”) was used. The crosslinked region around 5'-ETS bases 100–140, which was found also in the untagged control, is marked with an asterisk. **(C)** and **(D)** The two main regions of the 5'-ETS (A53-C96 and A192-C235) that were crosslinked to His_6_-Utp4 are shown together the number of mutations per base. The respective 5'-ETS sequence is depicted below. Mutational hot spots G66 and A220 colored in red.

The analysis of conserved surface patches of Utp4 revealed positively charged regions on top of β-propeller-1 and aside of β-propeller-2 that might serve as RNA binding regions. As Utp4 was recently shown to interact with the 5'-ETS region within the 35S pre-rRNA in *Saccharomyces cerevisiae* [23], we set out to map the respective regions in the 5'-ETS from *Chaetomium thermophilum*. Although the 5'-ETS is poorly conserved throughout eukaryotes, a similar fold with several stem-like RNA helices has been predicted for yeast as well as for *Chaetomium thermophilum*, where it comprises 587 nucleotides [9]. In order to identify direct RNA-protein interactions of Utp4, we performed *in vitro* RNA-protein UV crosslinking and mapped the binding regions within the target RNA by subsequent cDNA deep sequencing (CRAC) [16]. When we co-expressed the 5'-ETS together with the UTP-A factors in yeast and isolated the UTP-A complex by split-tag tandem affinity purification as reported before [12], we found that the 5'-ETS was co-purifying with this reconstituted 90S module (data not shown). Accordingly, we assembled and purified a similar 5'-ETS/UTP-A RNP carrying His_6_-tagged Utp4, which was used for *in vitro* UV crosslinking analysis (S3 Fig). Alignments of the reads obtained from deep sequencing analysis revealed that His_6_-Utp4 predominantly crosslinked to two distinct sites, whereas an untagged Utp4 control did not show such hits (Fig 4A and 4B). One RNA binding region was found at the 5'-end of the 5'-ETS (A53-C96), whereas the other was found further downstream (A192-U281). Notably, the second Utp4 interaction site overlaps with the 5'-ETS sequence G244-G254, which in the 90S pre-ribosome has been predicted to hybridize to the 3'-hinge region of the U3 snoRNA [9]. Sequencing also allowed identification of mutations (deletions or substitutions) within the co-purifying 5'-ETS fragments, which are indicative of bases that were actually crosslinked to Utp4. Among these we found two bases, G66 and A220, which turned out to be hotspots for mutations within the respective Utp4 crosslinking regions (Fig 4C and 4D).

### Docking of Utp4 into the 90S pre-ribosome

**Fig 5:**
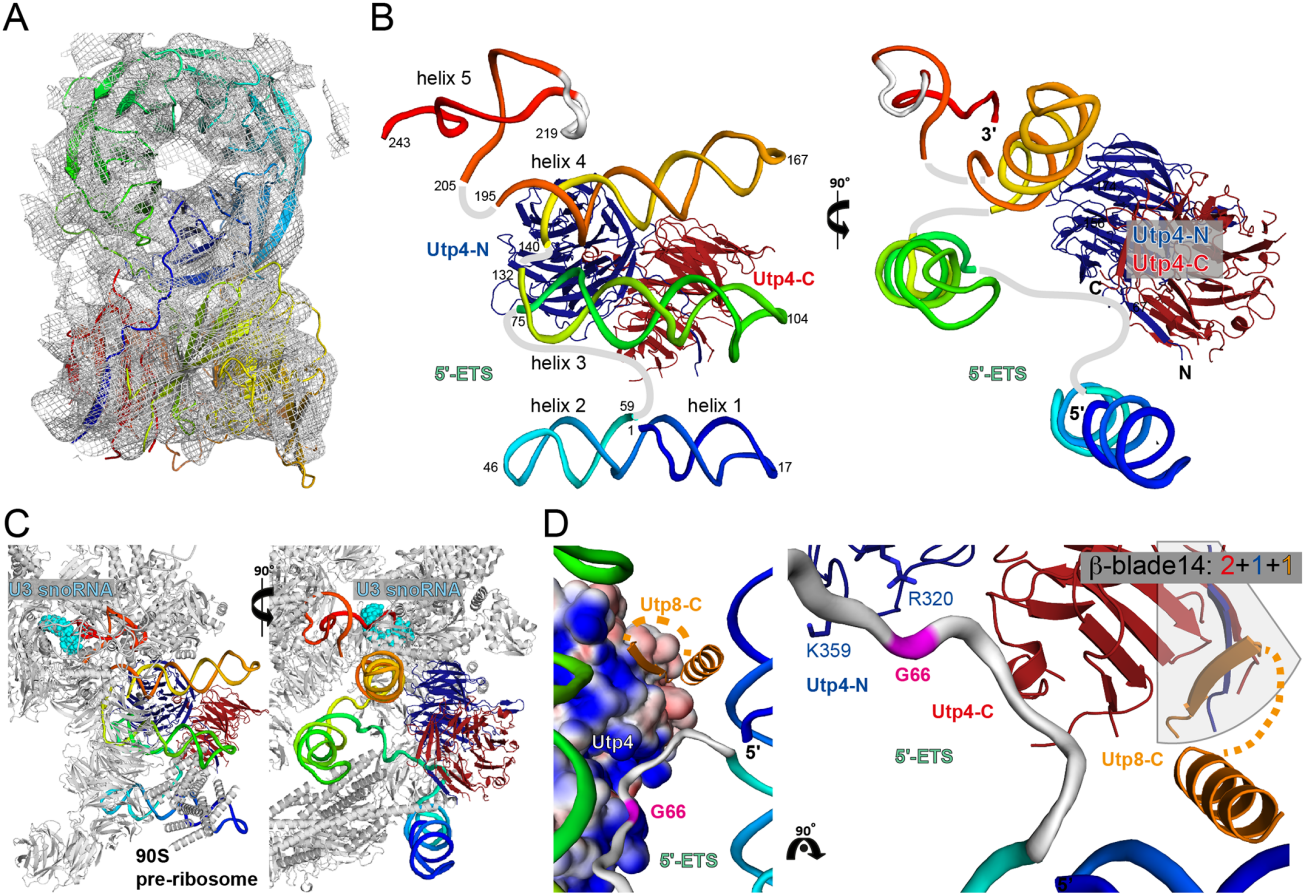
Utp4 in context of the 5'-ETS and 90S pre-ribosome. **(A)** The crystal structure of Utp4 (rainbow colours) placed into its cryo-EM density (overall 7.3 Å resolution of the particle) in context of the 90S pre-ribosome [9]. The density (grey mesh) is contoured at a 3σ level. The Utp4 structure fits in the EM-density as rigid body validating the relative propeller orientation and loop conformations as seen in the crystal structure in the physiological context. **(B)** Utp4 (blue: propeller 1 (Utp4-N); red: propeller 2 (Utp4-C)) in context of the entire 5'-ETS *de novo* modeled (rainbow) in the cryo-EM density. RNA helices and nucleotides at special positions are given. The 5’-end is hidden in the continuous stack of RNA helices 1 and 2. Single stranded RNA-parts are indicated by connecting lines (grey). **(C)** Utp4/5'-ETS in context of a close-up of the UTP-A complex as part of the entire 90S pre-ribosome complex (grey). The region of U3 snoRNA base-paring at the 3'-end of the 5'-ETS (beyond nucleotide 243) is highlighted in cyan. **(D)** Utp4/5'-ETS/Utp8 interaction around nucleotide G66 (magenta) identified as major contact point by CRAC analysis. Left panel: Utp4 is indicated by surface potential map (±5 *k*_*B*_T/e, blue positive). The C-terminus of Utp8 (Utp8-C, end of predicted α-helix and β-strand, no sequence modeled) are given in orange. The α-helix is *de novo* placed as ideal helix in the cryo-EM density, whereas the β-strand is taken from the artificial strand 14D of the X-ray structure (connection given as dashed lines is unclear). Right panel: Model for the Utp8-C interaction with Utp4. The Velcro-closed ‘2+1+1’ blade 14 is completed *in trans* by Utp8 and G66 binds to the positive patch (indicated by R^344^ and K^383^) in the Utp4-N/Utp4-C interface.

The crystal structure of Utp4 revealed the special orthogonal arrangement of the N-terminal β-propeller 1 in respect to β-propeller 2 and the Velcro-closure of the C-terminal blade 14 by the N-terminal β-strand of Utp4. We therefore asked, if this crystallographic finding is valid in the context of the early 90S pre-ribosome structure as solved recently at 7.3 Å resolution by single particle cryo-EM techniques [9]. Indeed, the Utp4 structure could be placed by rigid-body docking without any further adjustments and perfectly fits its previously assigned cryo-EM density (Fig 5A). Specific extensions from the propellers as the interacting protruding loops can be clearly localized within the density, however, the long unstructured C-terminal insertion appears also disordered within the pre-ribosomal complex. Utp4 is positioned in the center of the UTP-A complex and scaffolds large parts of the 5'-ETS.

In order to analyze the Utp4 structure within the complex, we *ab initio* built the 5'-ETS from its very 5'-end to the binding site for the U3 3'-hinge (starting at nucleotide 244) where the RNA forms a double-helix with U3 snoRNA (Fig 5B and 5C and S4 Fig). Although the resolution is limited, the double helical parts could be clearly assigned, guided by secondary structure predictions and previous biochemical data [23], and thus nucleotides in the connecting single stranded regions could be approximately placed. Strikingly and hitherto not known, helices 1 and 2 are coaxially stacked to form a single unit with the 5'-terminus of the RNA being hidden in the center of the merger. The distal tetranucleotide loop (tetraloop) of helix 2 is fixed by another β-propeller (Utp17, see below). Helices 3 to 5 match as expected from secondary structure predictions, and internal asymmetric bulges cause slight bending of the helices. The proximal ends of helices 3, 4, and 5 are in close proximity and tied together on the same β-propeller (Utp15, see below). Helix 5 within the 90S pre-ribosome is directly adjacent to the binding site for the U3 3'-hinge (Fig 5C).

Utp4 interacts with two regions of the 5'-ETS. From all RNA helices only helix 4 (nucleotides around 144 and 187) binds to short Utp4-loops originating from the N-terminal β-propeller 1 (Fig 5B). As these loops are unstructured in the Utp4 crystal structures, the interaction cannot be detailed any further. In the second interaction, Utp4 binds to the inter-helical single-stranded region (approx. nts 62–67) between helices 2 and 3 of the 5'-ETS. Most importantly, the nucleotide G66 identified as direct contact partner with Utp4 in our CRAC data, perfectly matches to the 5'-ETS model (Fig 5D). Although the exact mode of interaction has to be determined at higher resolution, it is evident that the binding surface for the single-stranded 5'-ETS region around G66 corresponds to the highly-conserved, positively charged surface patch of Utp4 located next to the β-propeller interface of Utp4 and the Velcro-closed blade 14 at the protein termini (Fig 2D).

The placement of Utp4 into the cryo-EM density also allows for the modeling of the complete closure of blade 14 by a UTP-A component *in trans*. Previous biochemical data have shown that the Utp4 C-terminal region interacts with Utp8. C-terminal truncations of Utp4 that remove blade 14 result in a loss of the Utp4-Utp8 interaction and the assembly of the 90S pre-ribosome, and in severe growth defects in yeast [10]. With this knowledge and the structural restraints at hand, we were able to deduce a first molecular model for the Utp4-Utp8 interaction involving blade 14 (Fig 5D). In this model, the extended Utp8 C-terminal α-helix emanating from the so-called ‘tetramer’ of the UTP-A complex crawls along the merged 5'-ETS helices 1 and 2 and directly points towards the blade. Theoretically, the extension could also belong to Utp5 (according to the Utp4-Utp5 interaction in yeast, [25]), however in contrast to the interaction with Utp8 there is no biochemical evidence for a Utp4-Utp5 interaction in *Chaetomium thermophilum*. Moreover, a single β-strand C-terminal to the extended α-helix is predicted for Utp8 (S2 Fig) that would localize next to the blade. We thus postulate, that the articifial β-strand from the TEV sequence present in our crystallized Utp4 construct mimics the physiological Velcro-closure *in trans* by Utp8. While the current data do not allow for discrimination whether this β-completion occurs parallel or antiparallel, the importance of forming a complete blade perfectly agrees with all biochemical and *in vivo* data. Taken together, Utp4 blade 14 is a binding site for Utp8 and the 5'-ETS.

## Discussion

While late assembly and maturation intermediates of the small and large ribosomal subunit have been studied in great detail, relatively little is known about the early co-transcriptional events during 90S pre-ribosome formation. As part of our attempts to decipher the very early steps of ribosome biogenesis we therefore focused on the UTP-A complex and especially on the Utp4 protein. From a previous cryo-EM study [9], it was evident that the double-propeller protein Utp4 plays a central role in organizing the UTP-A complex within the 90S pre-ribosome.

The high resolution crystal structure of the fungal Utp4 protein now reveals the two propellers to be rigidly linked and fixed in a tangential arrangement, as well as an unusual Velcro-closure of the last propeller-blade at a conserved and basic surface patch near the Utp4 termini. Although this unusual parallel complementation of the last blade could have been an artefact of our cloning strategy, it apparently represents a specific protein-binding site for Utp8 within the UTP-A complex as deduced from interaction studies [12, 23, 25, 26] and placing the Utp4 structure in a cryo-EM density of the 90S pre-ribosome. Within UTP-A, Utp4 and Utp17 present double-propellers. Although Utp17 also reveals direct propeller-interactions the arrangement is different and thus a general double-propeller architecture cannot be deduced.

For structural and functional characterization of Utp4 within the UTP-A complex, we first built a model of the 5'-ETS (nucleotides 1 to 243) based on the available cryo-EM density [9]. In order to validate our model biochemically, we mapped the 5'-ETS contacts by *in vitro* UV crosslinking (CRAC). The analysis showed that within the reconstituted 5'-ETS/UTP-A complex, Utp4 binds to 5'-ETS at two distinct sites. The RNA binding region located close to the 5'-end of the 5'-ETS (A53-C96) perfectly fits our model of the 5'-ETS, although it had not been included in first instance as building-restraint. The protein-RNA interaction at nucleotide G66 matches exactly. The first crosslink site is also in agreement to UTP-A CRAC data recently reported in yeast, in which Utp4 was crosslinked to the yeast 5'-ETS around bases 70–90 [23]. In contrast to our data, Hunziker and colleagues did not assign a second 5'-ETS region further downstream for Utp4 interaction. However, it has to be taken into account that our CRAC analysis was perfomed with the UTP-A complex bound to 5'-ETS *in vitro*. Within the entire 90S pre-ribosome, the identified second cross-link site is most likely shielded by other components such as the UTP-B, Mpp10 and U3 snoRNP modules. The UTP-B factor Pwp2 has been proposed to bind to the 5'-ETS region that forms a RNA hetero-duplex with the U3 snoRNA [9], which is close to our second RNA interaction region of Utp4.

**Fig 6:**
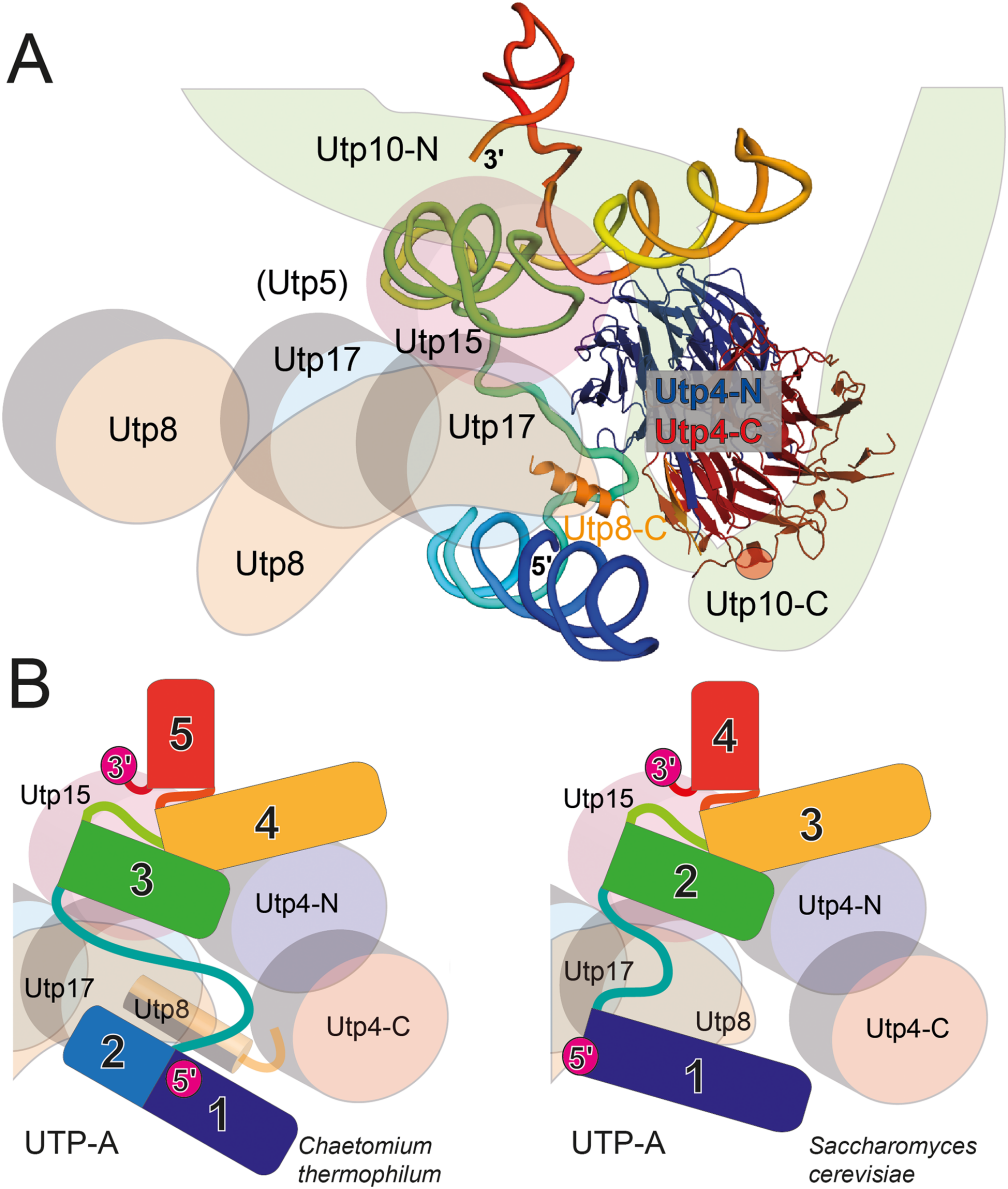
The UTP-A complex from *Chaetomium thermophilum*. **(A)** Scheme showing the spatial assembly of the fungal UTP-A complex including the Utp4 X-ray structure and the EM-modeled 5'-ETS. The propellers of remaining UTP-A proteins (Utp8, Utp15, and 2× Utp17) are placed according previous biochemical and EM-studies. The α-solenoidal parts (including whole Utp5) are not included. The entire Utp10 molecule turning around Utp4 is interpreted as also the very C-terminus (atomic model) of Utp8 next to the Velcro-closure of Utp4. The position of the disease-modified arginine in human Utp4 in the interface to Utp10 is highlighted within a red sphere. **(B)** Comparison of the UTP-A complexes from *Chaetomium thermophilum* (left panel; [9]) and *Saccharomyces cerevisiae* (right panel; [27, 28]). While the overall architecture is conserved, the 5'-end of the RNA shows a different arrangement. In addition, the Upt8-Utp4 contact is not visible in the yeast structures.

Based on the previously solved 90S cryo-EM structure [9] and our Utp4/5'-ETS structures we propose a first scheme for the entire UTP-A complex (Fig 6). Utp4 acts as a central organizer and forms extensive contacts with the linker between the N-terminal (not interpreted previously) and C-terminal solenoidal parts of Utp10. The huge Utp10 protein seemingly wraps around Utp4. Noteworthy, the disease relevant arginine residue (R565), mutated in the human homolog in patients suffering from NAIC (North American Indian childhood cirrhosis)[11], locates to the Utp4-Utp10 interface. Furthermore, Utp4 interacts with the C-terminal region of Utp8. Direct interactions are also established between Utp4 and β-propellers of Utp15 and Utp17. Utp4 might also contact other helical or unstructured parts of Utp5, Utp15, or Utp17. As atomic models of these proteins do not exist, detailed protein-protein interactions cannot be deduced yet.

An intact Utp4 C-terminus is required for cell growth and maturation of the 18S and 25S rRNAs [10]. In this study, different truncations at the Utp4 C-terminus were designed that resulted in severe growth defects in yeast. According to our Utp4 structure, these truncations would cause unfolding of the second β-propeller 2 and likely protein degradation. However, a C-terminal deletion introduced after the last parallel β-strand in blade 14 still allowed growth in yeast, pointing to the importance of Utp4 Velcro-closure for *in vivo* function. It has been proposed that Utp8 interacts with the C-terminus of Utp4 and that also this interaction is essential for the assembly of the 90S pre-ribosome [10]. Utp8 only interacted with full-length Utp4 or with truncations keeping blade 14, again validating our Utp4-Utp8 model.

Recently also cryo-EM structures of the yeast 90S pre-ribosome were published at higher resolutions [27, 28]. In these studies the 5'-ETS was not fully traced. Interestingly, helix 1 of the 5'-ETS in yeast is longer and corresponds to the merger of helices 1 and 2 in *Chaetomium thermophilum*, although the orientation of the helical stems is different (Fig 6B and S5 Fig). However, the 5'-ends in both RNAs are protected from nuclease attack either by internal helical stacking or, although not built in the yeast EM structure [27], by interaction with Utp17. Whether 5'-end burial is a general theme in 5'-ETS protection has to be investigated in the future. Due to the lack of any high resolution structure for any UTP-A double-propeller, Utp4 and Utp17 have been mixed-up in the study by Chaker-Margot et al. [27], whereas in the study by Sun et al. [28] only one β-propeller domain of Utp4 has been correctly placed. Our Utp4 structure fits the corresponding electron densities in both yeast 90S structures, highlighting the conformational conservation of fungal and probably all Utp4 homologs.

Taken together, the integration of our high-resolution Utp4 structure and RNA cross-linking data into the low resolution 90S cryo-EM structure allowed for the first description of the overall architecture of the earliest assembly in ribosome biogenesis. The rigid Utp4 double-propeller is found to orchestrate both, proteins of the UTP-A complex and the 5'-ETS RNA, and our detailed model for the 5'-ETS hints at important RNA features like 5'-end protection. Eukaryotic ribosome biogenesis depends on the highly dynamic and yet precisely coordinated interplay of many factors. With still only a few structures of intermediates at hand, we only begin to understand the underlying complex interaction networks. Evidently, higher resolution EM-studies of the 90S pre-ribosome have to be awaited for the final validation and completion of this first assembly intermediate.

## Accession numbers

Coordinates and structure factors are deposited in the RCSB protein data bank (PDB) with the accession number 5N1A. Sequencing data have been deposited at NCBI GEO with the accession number GSE98331.

## Supporting information

S1 Fig Interaction of β-strand 1D and *Chaetomium thermophilum* specific insertion. (**A**) The β-strand 1D of β-propeller 1 packs against the top of β-propeller 2, making multiple hydrogen bonds. (**B**) A *Chaetomium thermophilum* specific insertion in β-propeller 2 (between β-strands 13C and 13D, residues 717 to 825) is not resolved in the crystal structure.
(TIF)

S2 Fig Multiple sequence alignment of Utp4 and Utp8. **(A-B)** Multiple sequence alignment of N- and C-terminus of Utp4. **(C)** Multiple sequence alignment of C-terminus of Utp8 (Nol11 in metazoans), the predicted β-strand is shown in orange. Sequences of Utp4 and Utp8 of *Chaetomium thermophilum* (ct), *Saccharomyces cerevisiae* (sc), *Xenopus laevis* (xl), *Homo sapiens* (hs), and *Mus musculus* (mm) were aligned with Clustal Omega. Visualization and overlay of secondary structures was performed with ESPRIPT. Highly conserved residues are highlighted (red).
(TIF)

S3 Fig Assembly of the *Chaetomium thermophilum* UTP-A/5’-ETS RNP and *in vitro* CRAC analysis of *ct*Utp4. **(A)** Schematic representation of the yeast high-copy plasmid, from which the 5’-ETS rRNA (587 nt) was transcribed including a shortened 5’-UTR (12 nt) and a Cyc1 terminator-derived 3’ UTR (~200 nt). Transcription was driven by the galactose-inducible *GAL1* promoter, in which bases 345–472 of the promoter sequence have been deleted. RNA polymerase II start site is indicated by position +1. **(B)** Overview of the experimental procedure carried out for the CRAC analysis of Utp4. For further details see the Material and methods section. **(C)** Co-expression and affinity purification of the reconstituted UTP-A complex (via proteinA-TEV-*ct*Utp10) from a yeast strain, in which the 5’-ETS rRNA is transcribed from the construct described in A. Shown are the GST-TEV eluates (untagged Utp4 and His_6_-Utp4, respectively), analyzed by SDS-PAGE and Coomassie staining. Proteins were labeled according to previous mass spectrometry analysis of highly similar preparations. **(D)** NiNTA eluates of indicated samples (shown in C) after *in vitro* UV crosslinking and limited RNase digestion, resolved by SDS-PAGE. Utp4 crosslinked to radiolabelled RNA (marked at the right) was visualized by scanning the membrane in a PhosphorImager.
(TIF)

S4 Fig The Utp4/5’-ETS interaction in context of the 90S pre-ribosome. **(A)** The Utp4/5’-ETS complex shown with electron density (rRNA: grey, Utp4: blue; contoured at 2 σ) as derived from the cryo-EM reconstitution of the entire 90S pre-ribosome complex (1). While the RNA-helices are well defined, the single-stranded regions are only vaguely traceable. **(B)** The charged Utp4 interaction with the 5’-ETS. Utp4 is highly positively charged (blue, shown on surface potential +5 *k*_*B*_T/e) placing the RNA-helices 3 and 4 and single strand regions along both propellers. The single-stranded RNA region between helices 1 and 2 includes nucleotide G66 (magenta) in the propeller interface.
(TIF)

S5 Fig Superposition of UTP-A sub-complexes from *Chaetomium thermophilum* (ct) and *Saccharomyces cerevisiae* (sc). The Utp4/Utp8-C/5’ETS sub-complex of *Chaetomium thermophilum* placed in the cryo-EM density of the pre-90S particle (1) is superposed onto the equivalent structure from yeast (2) (based on Utp4 only). The rudimental-built RNA-helices from yeast 5’-ETS are given (magenta) highlighting the similar overall architecture of the 5’-ETS. The C-terminus of the shorter *sc*Utp8 homolog (rainbow colours) can be connected (dashed lines) to the extended C-terminus of *ct*UTP8 (orange) responsible for Velcro-closure of *ct*UTP4.
(TIF)

S1 Table List of constructs used in this study. (DOCX)
