# Supplementary figures and images for: Structural basis for 5'-ETS recognition by Utp4 at the early stages of ribosome biogenesis
Source: PLoS One. 2017 Jun 2;12(6):e0178752. doi: 10.1371/journal.pone.0178752 (PMC5456268; doi:10.1371/journal.pone.0178752)

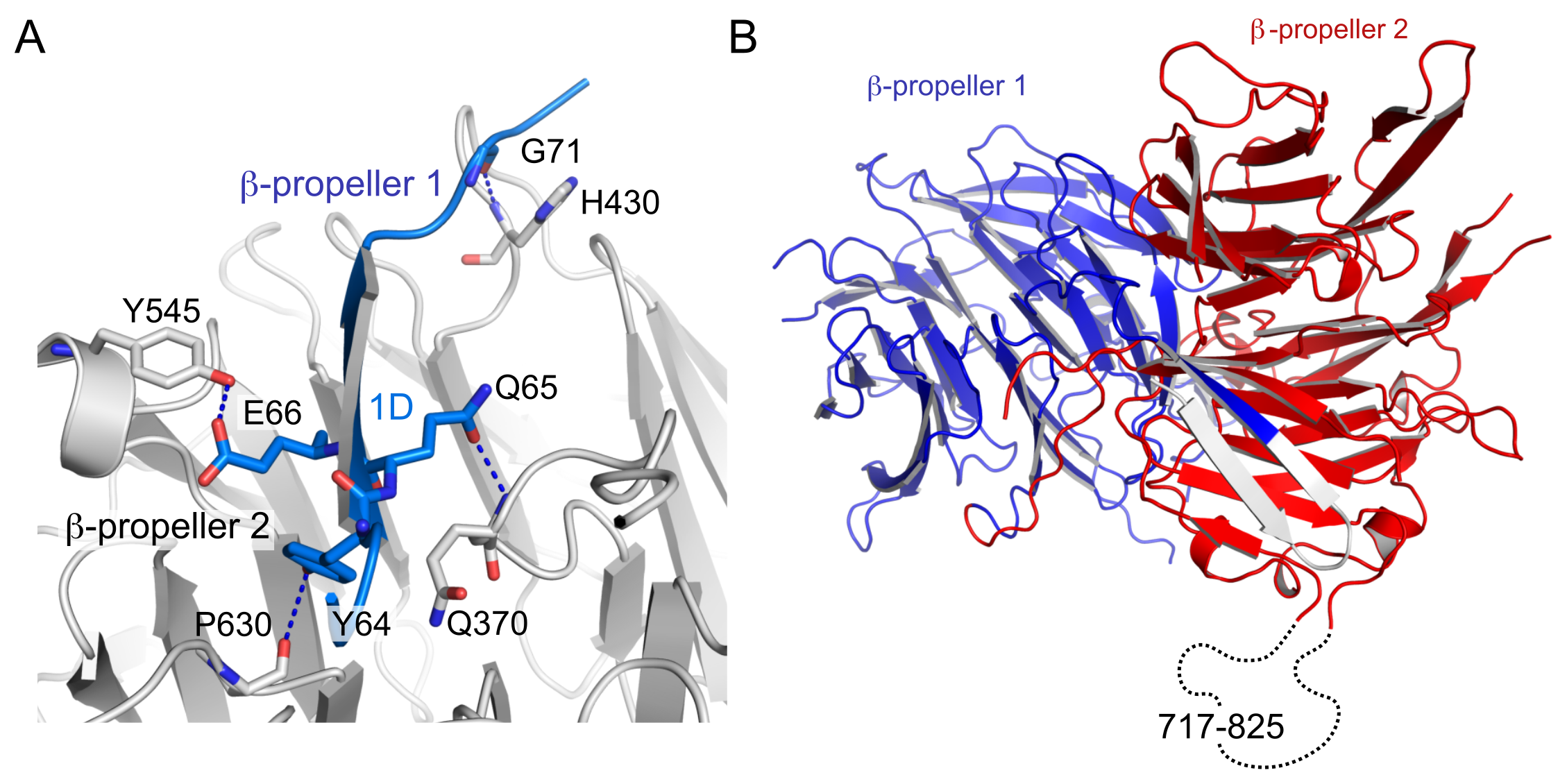

Supplement: S1 Fig — (A) The β-strand 1D of β-propeller 1 packs against the top of β-propeller 2, making multiple hydrogen bonds. (B) A Chaetomium thermophilum specific insertion in β-propeller 2 (between β-strands 13C and 13D, residues 717 to 825) is not resolved in the crystal structure. (TIF) [file pone.0178752.s001.tif]

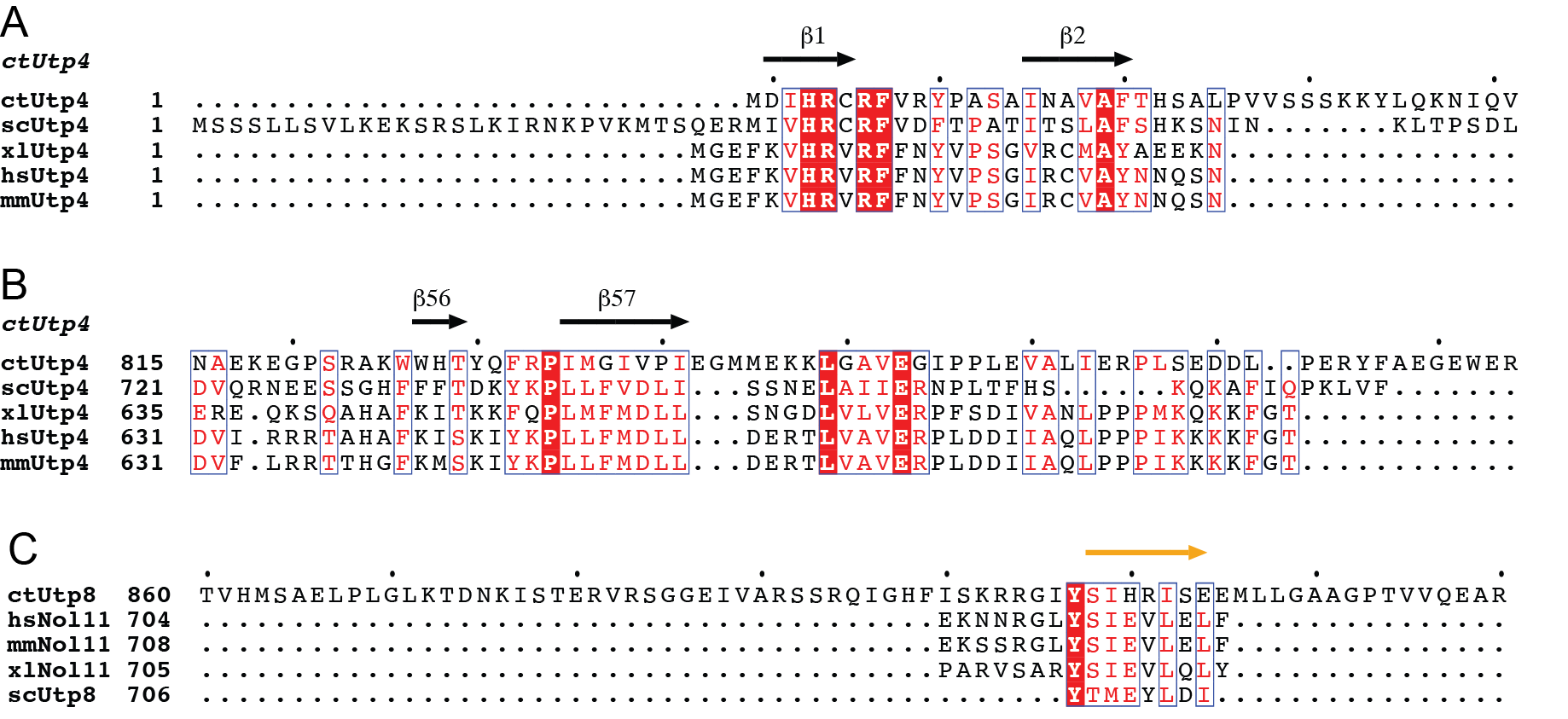

Supplement: S2 Fig — (A-B) Multiple sequence alignment of N- and C-terminus of Utp4. (C) Multiple sequence alignment of C-terminus of Utp8 (Nol11 in metazoans), the predicted β-strand is shown in orange. Sequences of Utp4 and Utp8 of Chaetomium thermophilum (ct), Saccharomyces cerevisiae (sc), Xenopus laevis (xl), Homo sapiens (hs), and Mus musculus (mm) were aligned with Clustal Omega. Visualization and overlay of secondary structures was performed with ESPRIPT. Highly conserved residues are highlighted (red). (TIF) [file pone.0178752.s002.tif]

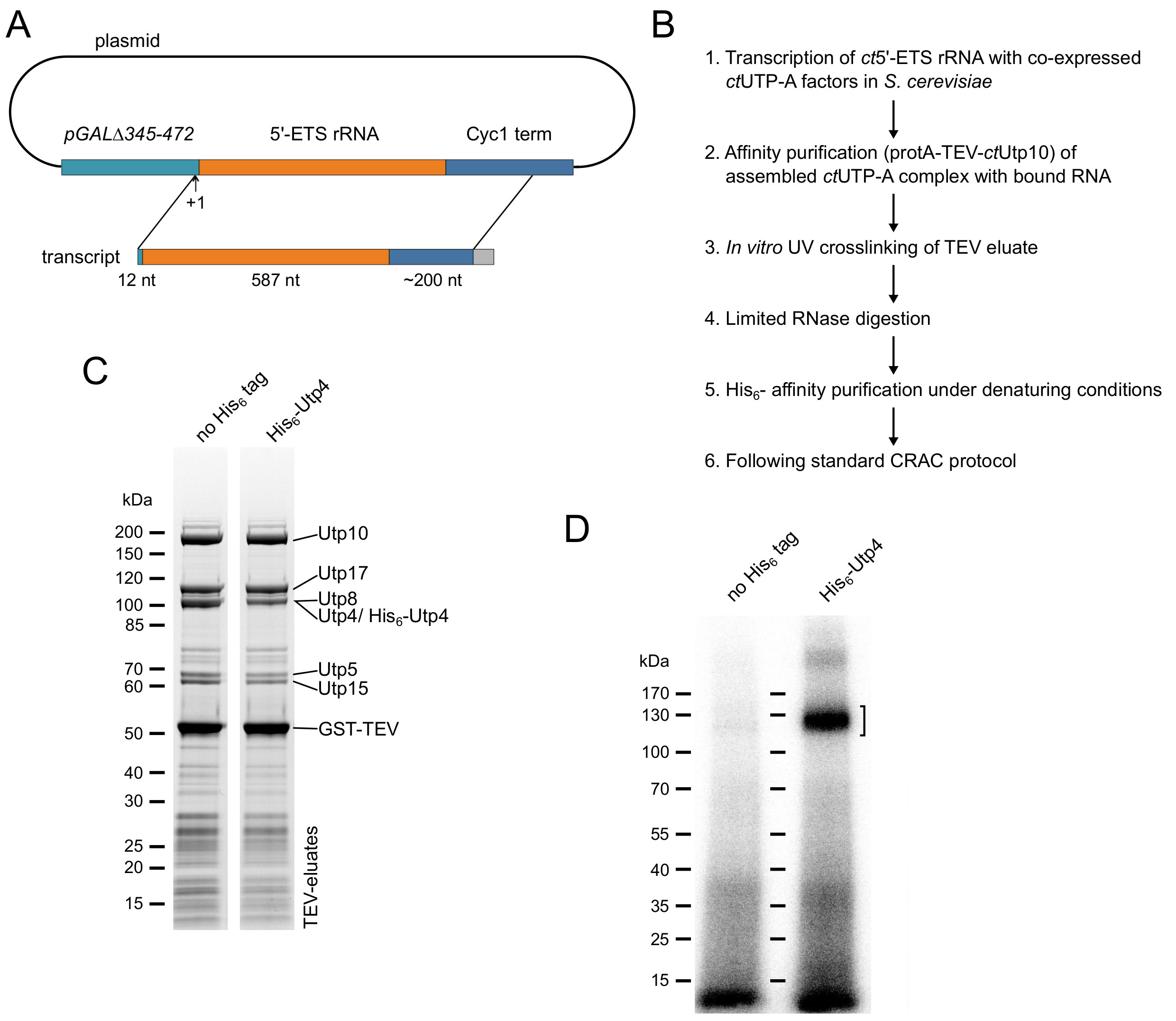

Supplement: S3 Fig — (A) Schematic representation of the yeast high-copy plasmid, from which the 5’-ETS rRNA (587 nt) was transcribed including a shortened 5’-UTR (12 nt) and a Cyc1 terminator-derived 3’ UTR (~200 nt). Transcription was driven by the galactose-inducible GAL1 promoter, in which bases 345–472 of the promoter sequence have been deleted. RNA polymerase II start site is indicated by position +1. (B) Overview of the experimental procedure carried out for the CRAC analysis of Utp4. For further details see the Material and methods section. (C) Co-expression and affinity purification of the reconstituted UTP-A complex (via proteinA-TEV-ctUtp10) from a yeast strain, in which the 5’-ETS rRNA is transcribed from the construct described in A. Shown are the GST-TEV eluates (untagged Utp4 and His6-Utp4, respectively), analyzed by SDS-PAGE and Coomassie staining. Proteins were labeled according to previous mass spectrometry analysis of highly similar preparations. (D) NiNTA eluates of indicated samples (shown in C) after in vitro UV crosslinking and limited RNase digestion, resolved by SDS-PAGE. Utp4 crosslinked to radiolabelled RNA (marked at the right) was visualized by scanning the membrane in a PhosphorImager. (TIF) [file pone.0178752.s003.tif]

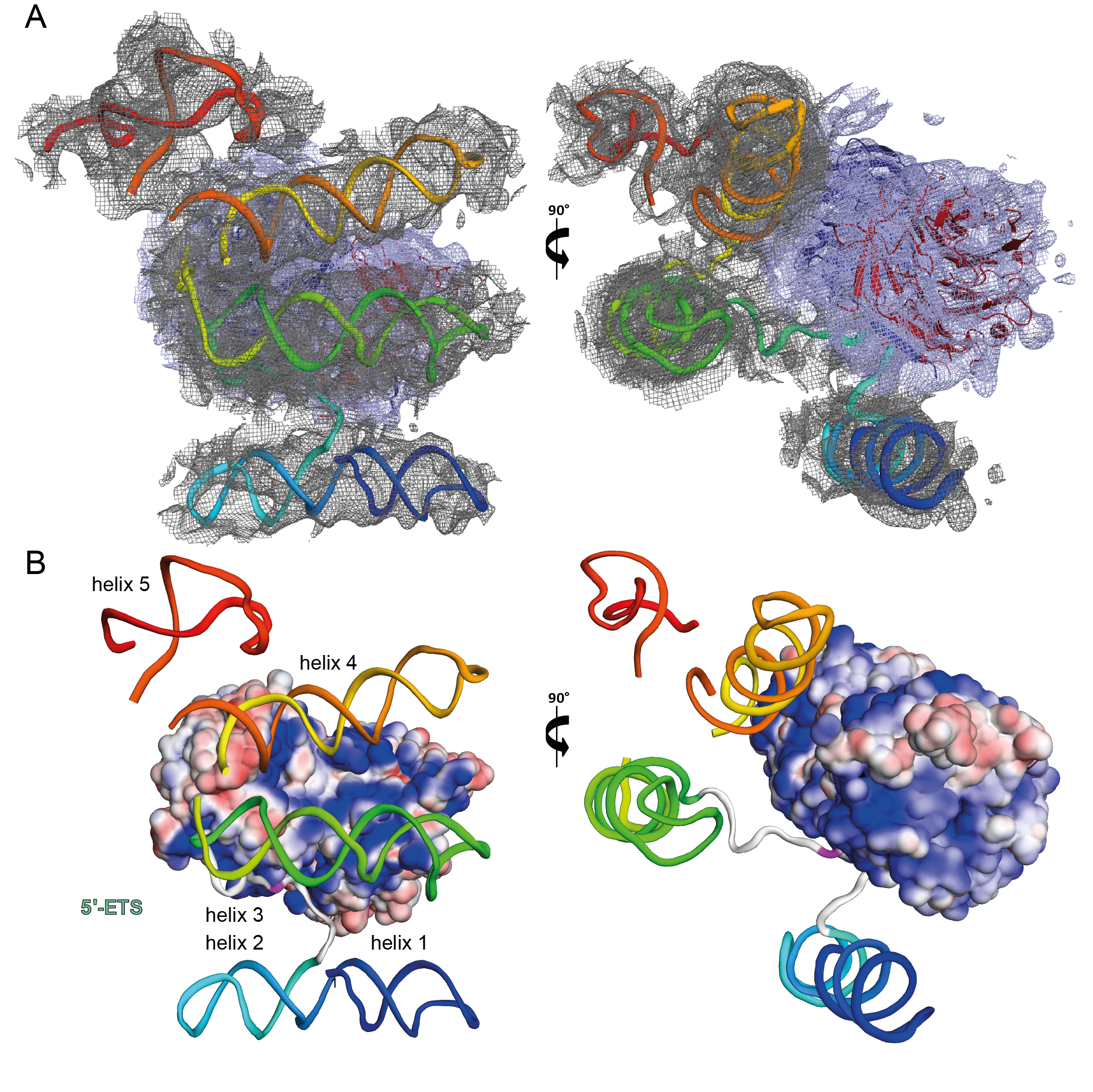

Supplement: S4 Fig — (A) The Utp4/5’-ETS complex shown with electron density (rRNA: grey, Utp4: blue; contoured at 2 σ) as derived from the cryo-EM reconstitution of the entire 90S pre-ribosome complex (1). While the RNA-helices are well defined, the single-stranded regions are only vaguely traceable. (B) The charged Utp4 interaction with the 5’-ETS. Utp4 is highly positively charged (blue, shown on surface potential +5 kBT/e) placing the RNA-helices 3 and 4 and single strand regions along both propellers. The single-stranded RNA region between helices 1 and 2 includes nucleotide G66 (magenta) in the propeller interface. (TIF) [file pone.0178752.s004.tif]

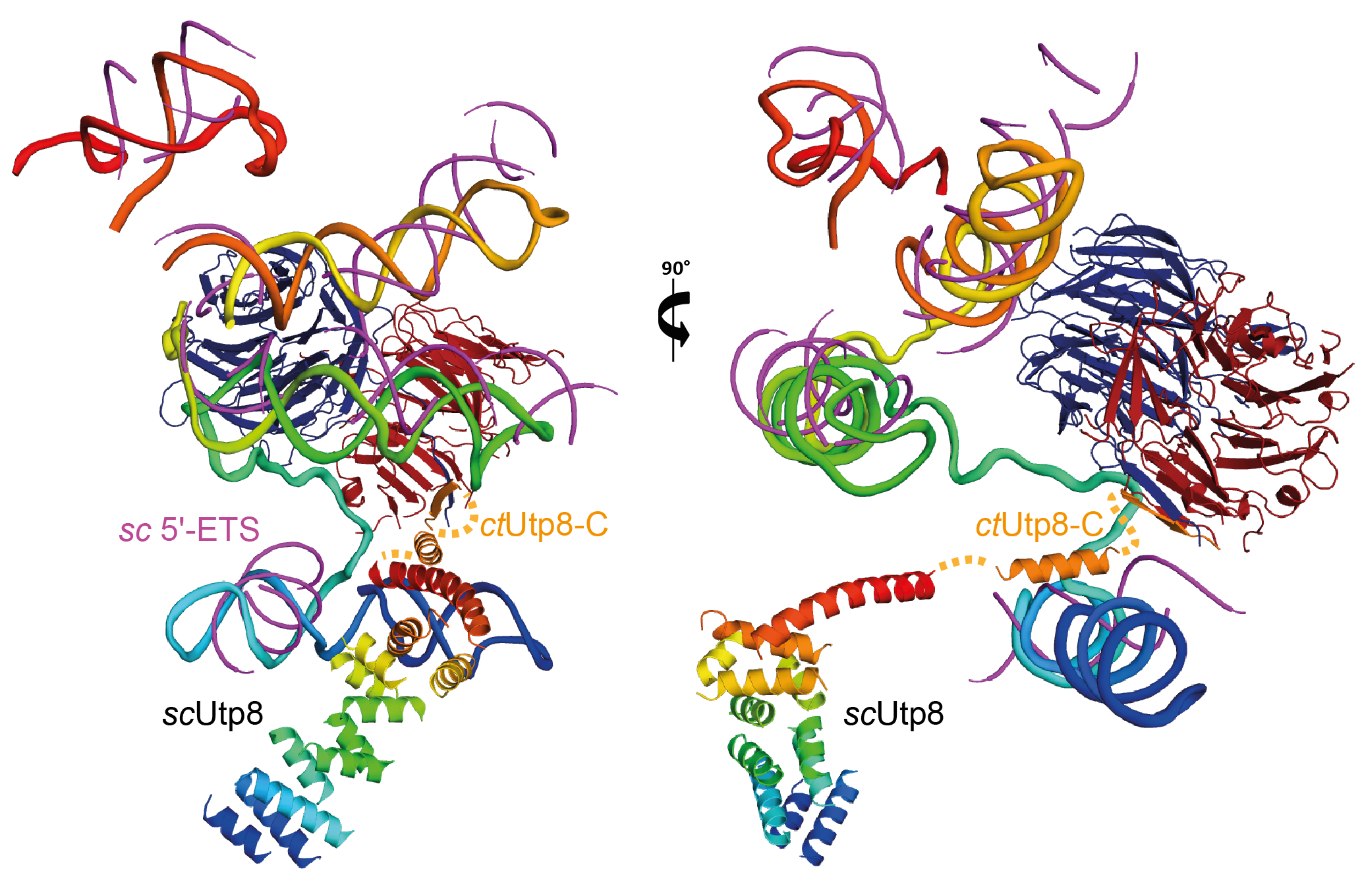

Supplement: S5 Fig — The Utp4/Utp8-C/5’ETS sub-complex of Chaetomium thermophilum placed in the cryo-EM density of the pre-90S particle (1) is superposed onto the equivalent structure from yeast (2) (based on Utp4 only). The rudimental-built RNA-helices from yeast 5’-ETS are given (magenta) highlighting the similar overall architecture of the 5’-ETS. The C-terminus of the shorter scUtp8 homolog (rainbow colours) can be connected (dashed lines) to the extended C-terminus of ctUTP8 (orange) responsible for Velcro-closure of ctUTP4. (TIF) [file pone.0178752.s005.tif]
