# Supplementary material for: Structural basis for 5'-ETS recognition by Utp4 at the early stages of ribosome biogenesis
Source: PLoS One. 2017 Jun 2;12(6):e0178752. doi: 10.1371/journal.pone.0178752 (PMC5456268; doi:10.1371/journal.pone.0178752)
Supplement: S1 Table — (DOCX) [file pone.0178752.s006.docx]

**S1 Table. List of constructs used in this study.**

| Construct | Notes | Source |
| --- | --- | --- |
| pET24d-His_6_-TEV-*ct*Utp4 | for protein expression in *E. coli*, f1 origin, T7 promoter, pBR322 origin, KanR, N-terminal 6×His-TEV tag | Baßler et al., 2016 (3) |
| YEplac112-pGAL-P1:Flag-*ct*Utp15-P2:*ct*Utp17 | for GAL-induced co-expression in yeast, 2µ, TRP1, PGAL1-10, TADH1, N-terminal Flag tag | Baßler et al., 2016 (3) |
| YEplac181-pGAL-P1:pA-TEV-*ct*Utp10-P2:*ct*Utp5 | for GAL-induced co-expression in yeast, 2µ, LEU2, PGAL1-10, TADH1, N-terminal proteinA-TEV tag | Baßler et al., 2016 (3) |
| YEplac195-pGAL-P1:*ct*Utp8-P2:*ct*Utp4 | for GAL-induced co-expression in yeast, 2µ, URA3, PGAL1-10, TADH1, no tags | Baßler et al., 2016 (3) |
| YEplac195-pGAL-P1:*ct*Utp8-P2:His_6_-*ct*Utp4 | for GAL-induced co-expression in yeast, 2µ, URA3, PGAL1-10, TADH1, N-terminal His_6_ tag | this study |
| pRS423-pGAL∆5’UTR-*ct*5’-ETS | for GAL-induced RNA transcription in yeast, 2µ, HIS3, PGAL1, TCYC1, *ct*5’-ETS(1-587nt), GAL1∆345-472nt truncated promoter | this study |

1. Kornprobst, M., Turk, M., Kellner, N., Cheng, J., Flemming, D., Kos-Braun, I., Kos, M., Thoms, M., Berninghausen, O., Beckmann, R. *et al.* (2016) Architecture of the 90S Pre-ribosome: A Structural View on the Birth of the Eukaryotic Ribosome. *Cell*, **166**, 380-393.

2. Chaker-Margot, M., Barandun, J., Hunziker, M. and Klinge, S. (2017) Architecture of the yeast small subunit processome. *Science*, **355**.

3. Bassler, J., Ahmed, Y.L., Kallas, M., Kornprobst, M., Calvino, F.R., Gnadig, M., Thoms, M., Stier, G., Ismail, S., Kharde, S. *et al.* (2016) Interaction network of the ribosome assembly machinery from a eukaryotic thermophile. *Protein Sci*.
